# Commentary: Fasting-Mimicking Diet Reduces HO-1 to Promote T Cell-Mediated Tumor Cytotoxicity

**DOI:** 10.3389/fonc.2017.00116

**Published:** 2017-06-02

**Authors:** Giovanni Li Volti, Roberto Avola, Manlio Vinciguerra

**Affiliations:** ^1^Department of Biomedical and Biotechnological Sciences, University of Catania, Catania, Italy; ^2^Center for Translational Medicine (CTM), International Clinical Research Center (ICRC), St. Anne’s University Hospital, Brno, Czechia

**Keywords:** fasting, chemotherapy, liver neoplasms, nuclear localization, chemoresistance

Heme oxygenase (HO)-1 is an evolutionarily conserved enzyme expressed in mammalian cells. This protein is the first and rate-limiting enzyme in heme catabolism, degrading heme to equimolar quantities of carbon monoxide (CO), free iron, and biliverdin; biliverdin later converts to bilirubin, while free iron is directly sequestered by ferritin (1). HO-1 is expressed at low levels under basal conditions, and it is induced by polyphenols and a variety of stimuli such as inflammation, oxidative stress, hyperoxia, and hypoxia (2). Such upregulation represents an intrinsic defense mechanism to maintain cellular homeostasis. In cancer cells, HO-1 is considered to play a major role as an essential survival factor, protecting against chemotherapy-induced reactive oxygen species increase. In particular, exposure to CO sensitized prostate cancer cells but not normal cells to chemotherapy (3). Similarly, CO treatment results in positive alterations of tumor microenvironment impeding lung cancer growth through the modulation of macrophages (4). Interestingly, recent findings showed that other mechanisms not related to HO-1 enzymatic activity might be responsible for its antitumor and chemoresistance activities (5, 6). In particular, HO-1 nuclear translocation has been shown mediating, at least in part, some of these activities (7). Surprisingly, so far such non-enzymatic effects in cancer cells were only described for tumors of epithelial origin and some hematological malignancies. We read with great interest the elegant work of Di Biase et al. (8) in which the authors showed that fasting-mimicking diet (FMD) significantly reduced HO-1 expression in cancer cells (i.e., breast cancer and melanoma), whereas it increased the protein levels in normal cells. The authors further showed that this effect resulted in a significant increase in the number of cytotoxic CD8^+^ tumor-infiltrating lymphocytes. Such conclusions are based on the authors’ data showing that induction of HO-1 by pharmacological mean (i.e., hemin) or gene overexpression abolished the beneficial effect of FMD mediated HO-1 reduction on chemosensitivity. Interestingly, the authors stated in their manuscript that such external manipulation of HO-1 resulted only in a partial abolishment of the STS effects. In this respect, we would like to highlight authors’ data (Figure S5E) showing abundant HO-1 protein exclusively in the nuclear fraction in untreated control cells, and no signal at all upon STS, meaning that expression rather than nuclear translocation is affected. These data do suggest that nuclear HO-1 strongly, if not totally, contributes to 4T1 cells fitness, and may lead to the hypothesis that hemin may have a role not only in HO-1 upregulation and activation but also on its nuclear import. Consistently with this observation, previous published data demonstrated that hemin, a strong inducer of HO-1 expression and HO activity, can induce nuclear translocation of HO-1 in two different prostate cancer cells (9). Conversely, the authors also showed that HO-1 inhibitor ZnPP sensitized breast cancer cells (4T1) to cyclophosphamide under normal conditions *in vitro* (Figure 4G). It should be taken into due account that ZnPP is not a specific inhibitor of HO-1 activity since it results also in a significant inhibition of the constitutive isoform (i.e., HO-2) activity (10). In addition, ZnPP induce a significant increase of HO-1 protein expression due to intracellular heme overload, thus providing more substrate for the cleavage necessary for nuclear compartmentalization. Finally, this work further demonstrates the need of appropriate molecular tools to dissect the enzymatic function of HO-1 from its non-canonical functions.

## Author Contributions

GV, RA, and MV contributed to critical review of the literature and wrote the manuscript.

## Conflict of Interest Statement

The authors declare that the research was conducted in the absence of any commercial or financial relationships that could be construed as a potential conflict of interest.
